# Development of bioengineered SPIONs using *Carica monoica* extract: evaluation of antioxidant and anticancer activity of human cervical cancer cells (SiHa)

**DOI:** 10.1515/biol-2025-1258

**Published:** 2026-02-12

**Authors:** Xiyi Tian, Haiyuan Yang

**Affiliations:** He University, Hunnan District, Shenyang, 110163, China; Cervical Disease Clinic, Maternal and Child Health Care in Jiujiang City, Jiujiang, Jiangxi, 332000, China

**Keywords:** antioxidants, magnetic nanoparticles, SiHa cells, apoptosis, fluorescence

## Abstract

This study pioneers the green synthesis of SPIONs using a novel bioresource: *Carica monoica* leaf extract as a reducing and capping agent. This method not only emphasizes sustainability but also uniquely leverages the specific phytochemical profile of *C. monoica* to create a synergistic therapeutic platform. Comprehensive characterization using UV-vis, XRD, TEM, EDX, and FTIR, Zeta potential and VSM confirmed the successful creation of hexagonal, superparamagnetic nanoparticles with an average size of 30–50 nm. The biological activity of these CM-SPIONs revealed a dual and potent functionality. Multiple assays showed the nanoparticles have significantly enhanced, dose-dependent antioxidant activity, indicating a nano-bio synergy that amplifies the phytoconstituents’ effects. More importantly, this antioxidant potential translated into selective and potent anticancer efficacy against human cervical cancer (SiHa) cells. MTT assay revealed potent, dose-dependent cytotoxicity with a notably low IC_50_ value of 22 ± 0.7 μg/ml. Fluorescence microscopy using AO/EtBr and DAPI staining confirmed that the mechanism of cell death was predominantly apoptosis, providing initial mechanistic insight. Thus, CM-SPIONs function as an active nanotherapeutic agent, demonstrating the potential of novel plant biosystems to yield sustainable nanomedicines with dual antioxidant and pro-apoptotic efficacy.

## 1 Introduction

Cancer is a pervasive and life-threatening disease, with its global prevalence continuing to rise [1], 2]. Epidemiological studies indicate that approximately one in six women and one in five men will develop a fatal malignancy during their lifetime [3]. The high mortality rate associated with cancer can be attributed to two major factors: the limited specificity of treatments in targeting cancerous cells without harming healthy tissues and the development of resistance to therapeutic drugs [4]. According to recent global cancer reports, there were 18.1 million new cancer cases and 9.6 million cancer-related deaths, with lung cancer being the leading cause of mortality, followed by breast, colorectal, stomach, and liver cancers [5].

Cervical cancer (CC) is a prevalent malignancy of the female reproductive system, with its prevalence ranking second among gynaecologic cancers. It presents a significant risk to women’s health, with rising incidence and fatality rates globally [6]. Recent figures indicate that around 90 % of cervical cancer fatalities transpire in poor and middle-income nations, with projections suggesting a 50 % rise in yearly cervical cancer-related deaths by 2040, reaching 460,000 therefore ranking second to breast cancer in fatality rates [7]. Cervical cancer is one of the most frequent types of cancer affecting women worldwide. Women in developing countries are disproportionately affected by cervical cancer compared to those in wealthy nations [8]. Annually, around 350,000 new instances of cervical cancer are diagnosed, constituting 10 % of all malignancies in women [9]. Despite being preventable and treatable if discovered early, it remains a primary contributor to disability-adjusted life years among many individuals [10].

Chemotherapy is a prevalent intervention used to treat and combat cancer; yet, it entails certain drawbacks that may profoundly impact patients’ quality of life. Chemotherapy is successful in targeting and eliminating cancer cells, although it has its downsides. This encompasses several adverse effects, including nausea, exhaustion, alopecia, and immunosuppression, leaving patients susceptible to infections. Furthermore, chemotherapy may impact healthy cells, resulting in enduring health complications such as organ damage or secondary malignancies. Moreover, the emotional and psychological impact of the treatment procedure, along with the financial strain, may exacerbate overall patient suffering. Comprehending these disadvantages is crucial for controlling patient expectations and investigating alternative or adjunctive therapy alternatives [11]. This underscores the urgent need for the development of novel, targeted, and more biocompatible therapeutic strategies.

Antioxidant activity refers to the ability of certain compounds to neutralize harmful free radicals in the body. Free radicals are unstable molecules that cause oxidative stress, leading to cellular damage and contributing too many diseases and the ageing process. Antioxidants operate by giving electrons to free radicals, therefore stabilizing them and preventing further damage. This preventive method contributes to maintaining overall health and reducing the risk of chronic diseases, such as cardiovascular illnesses, cancer, and neurological conditions [12].

Nanotechnology has emerged as a transformative tool in oncology, offering innovative platforms for diagnosis, imaging, and targeted drug delivery [13]. Among various nanomaterials, superparamagnetic iron oxide nanoparticles (SPIONs) hold exceptional promise due to their unique magnetic properties, biocompatibility, and potential for surface functionalization [14]. Traditionally synthesized through physical and chemical methods, these processes often involve toxic reductants, high energy consumption, and generate hazardous byproducts, limiting their biomedical applicability and raising environmental concerns [15].

In this context, green synthesis utilizing plant extracts, microbes or other biological systems has gained considerable traction as a sustainable and eco-friendly alternative [16]. This approach leverages the rich reservoir of phytochemicals such as polyphenols, flavonoids, alkaloids, and terpenoids, which act as both reducing and stabilizing agents [17]. Beyond merely providing a “green” route, the phytoconstituents from the plant extract can cap the nanoparticles, potentially enhancing their stability, biocompatibility, and bioactivity. This creates a synergistic bio-nano interface where the biological activity of the phytochemicals is integrated with the unique physicochemical properties of the nanomaterial [18].

Superparamagnetic iron oxide nanoparticles (SPIONs) and their derivatives exhibit notable characteristics, including superparamagnetic properties, small dimensions, and high saturation magnetization, rendering them suitable for biological applications such as cancer cell detection [19], 20], methods of drug administration, and magnetic resonance imaging (MRI) of cells and tissues [21], 22]. SPION’s adaptability enables its utilization in several biomedical fields, including magnetic resonance imaging (MRI) as a contrast agent [23], drug administration [24], magnetic hyperthermia treatment (MHT) [25], and biosensing [26]. The direct application of SPION is limited by its tendency to accumulate in bodily fluids and its subsequent clearance by reticuloendothelial system (RES) cells [27], 28]. Moreover, bare SPION are susceptible to oxidation, leading to alterations in their magnetic and chemical characteristics. Consequently, it is essential to use protective coatings or vesicles to alleviate the limitations of uncoated SPION [27], 29], 30]. Research indicates enhanced characteristics with the integration of SPION in nanocarriers, including liposomes [31], 32] and polymeric nanoparticles [33]. Recent studies have evaluated SPIONs loaded with different medications using several drug delivery techniques. Abbas et al. [34] developed SPION-loaded lipid nanocarriers incorporated into a thermosensitive *in situ* gel, enabling tailored magnetic distribution to augment clonazepam concentration in the brain for the treatment of neurological disorders.

Critically, green-synthesized SPIONs are not merely inert carriers; they often exhibit intrinsic therapeutic properties. The capping biomolecules can confer significant antioxidant activity, enabling the nanoparticles to modulate oxidative stress a key factor in cancer progression and therapy resistance [35]. Furthermore, these nanoparticles have demonstrated direct anticancer effects through various mechanisms, including the induction of apoptosis, cell cycle arrest, and the generation of reactive oxygen species (ROS) within cancer cells [36]. The benefits of this approach are multifold: it is cost-effective, reduces environmental toxicity, improves biocompatibility, and can yield nanoparticles with enhanced and multifunctional bioactivity compared to their chemically synthesized counterparts.

*Carica monoica*, a less-explored species of the papaya family, is known in traditional medicine and is a rich source of bioactive compounds. However, its potential in the green synthesis of SPIONs and the subsequent biomedical application of the nanoparticles remains entirely unexplored. Therefore, this study aims to bridge this gap by developing a novel, one-pot green synthesis of SPIONs using an aqueous extract of *C. monoica* leaves. We hypothesize that the phytoconstituents in the extract will successfully synthesize and stabilize SPIONs, endowing them with combined antioxidant and anticancer properties. The synthesized *CM*-SPIONs were thoroughly characterized. Their antioxidant capacity was systematically evaluated using a battery of standard assays. Furthermore, their anticancer efficacy and putative mechanism of action were investigated against human cervical cancer (SiHa) cells through cytotoxicity, morphological, and fluorescence-based apoptotic assays. This work positions *C. monoica* as a novel bioresource for creating a sustainable, multifunctional nanotherapeutic agent with promising applications in cancer therapy.

## 2 Materials and methods

### 2.1 Chemicals and reagents

All chemicals and reagents used were of analytical grade. Ferric chloride hexahydrate (FeCl_3_·6H_2_O, ≥99 %) and ferrous chloride tetrahydrate (FeCl_2_·4H_2_O, ≥99 %) were procured from Sigma-Aldrich (USA) and used as the iron precursors for nanoparticle synthesis. Ammonium hydroxide (NH_4_OH, 25 %) was obtained from Merck (Germany) and used as a precipitating agent. For the assessment of antioxidant activity, the following reagents were used: 2,2-diphenyl-1-picrylhydrazyl (DPPH), 2,2′-azino-bis(3-ethylbenzothiazoline-6-sulfonic acid) (ABTS), potassium persulfate, ferric reducing antioxidant power (FRAP) reagent (containing 2,4,6-tris (2-pyridyl)-s-triazine (TPTZ)), iron (III) chloride, ascorbic acid, nitroblue tetrazolium (NBT) for the superoxide dismutase (SOD) assay, phenazine methosulfate (PMS), nicotinamide adenine dinucleotide (NADH), hydrogen peroxide (H_2_O_2_, 30 %), potassium ferricyanide, trichloroacetic acid (TCA), and Folin-Ciocalteu reagent. Gallic acid, quercetin, and butylated hydroxytoluene (BHT) were used as standard antioxidant compounds. For cell culture and anticancer assays, Dulbecco’s Modified Eagle Medium (DMEM), fetal bovine serum (FBS), penicillin-streptomycin antibiotic solution, and trypsin-EDTA were purchased from Gibco (Thermo Fisher Scientific, USA). The 3-(4,5-dimethylthiazol-2-yl)-2,5-diphenyltetrazolium bromide (MTT) assay kit was obtained from Sigma-Aldrich. For fluorescence microscopy and cell death analysis, acridine orange (AO), ethidium bromide (EtBr), 4′,6-diamidino-2-phenylindole (DAPI), and the reactive oxygen species (ROS) detection dye 2′,7′-dichlorodihydrofluorescein diacetate (DCFH-DA) were also sourced from Sigma-Aldrich. All aqueous solutions were prepared using deionized (DI) water (Milli-Q system, 18.2 MΩ cm).

### 2.2 *Carica monoica* collection and extraction

The leaves of *C. monoica* were harvested from the wild. The leaves of *C. monoica* were rinsed with tap water and then shade-dried at ambient temperature (28 ± 2 °C) for a duration of 10–15 days. The desiccated *C. monoica* leaves were pulverized using a blender mixer grinder. Five grams of dried papaya leaf powder are submerged in 50 ml of distilled water for a period of 30 min to 1 h to promote the softening of the leaves. Elevate the temperature of the mixture till it attains its boiling point. Once boiling, reduce the heat and let it to simmer for 30 min to 1 h. This enables the extraction of soluble compounds from the leaves. Allow the mixture to attain room temperature after boiling. Employ a fine mesh strainer or cheesecloth to separate the liquid extract from the solid bark pieces, and then filter the liquid using Whatman filter paper. Store the papaya leaf extract in sanitized, airtight containers in fridge for future use [37].

### 2.3 Synthesis and characterization of CM-SPIONs

Superparamagnetic iron oxide nanoparticles (SPIONs) were synthesized via a modified co-precipitation method, utilizing *C. monoica* leaf extract as a green reducing and capping agent. The procedure was optimized based on established protocols for plant-mediated synthesis and preliminary trials. A 0.1 M aqueous solution of ferric chloride hexahydrate (FeCl_3_·6H_2_O) was prepared by dissolving 1.35 g in 50 ml of deionized water. Similarly, a 0.05 M aqueous solution of ferrous sulfate heptahydrate (FeSO_4_·7H_2_O) was prepared by dissolving 0.695 g in 50 ml of deionized water. These concentrations and the molar ratio of Fe^3+^:Fe^2+^ (2:1) were selected to provide the stoichiometry (Fe^3+^/Fe^2+^ = 2/1) theoretically required for the formation of magnetite (Fe_3_O_4_). The 50 ml ferrous sulfate solution was added to a 250 ml three-neck round-bottom flask under constant mechanical stirring at 500 rpm. To this, the 50 ml ferric chloride solution was added dropwise over 5 min. Subsequently, 100 ml of the freshly prepared *C. monoica* leaf extract (10 % w/v) was introduced into the mixed iron ion solution. The 1:1 volume ratio of total iron solution to plant extract was chosen based on preliminary experiments, which indicated that this proportion provided sufficient phytochemicals for complete reduction and effective stabilization of the nanoparticles without excess unreacted organics. To initiate and control the precipitation reaction, the pH of the reaction mixture was carefully raised to 10–11 by the slow, dropwise addition of 2 M ammonium hydroxide (NH_4_OH) solution. The alkaline pH is critical for facilitating the hydrolysis and co-precipitation of iron oxides. Upon base addition, the solution color changed from pale yellow to an immediate dark brown/black, indicating the formation of iron oxide nanoparticles. The reaction mixture was then heated to 70 ± 2 °C and maintained under vigorous stirring for 4 h to allow for complete particle growth and stabilization by the phytoconstituents. After the reaction, the mixture was allowed to cool to room temperature. The synthesized nanoparticles (CM-SPIONs) were separated from the aqueous suspension by centrifugation at 10,000 rpm for 30 min. The pellet was washed thoroughly three times with deionized water and once with absolute ethanol to remove any unbound biological residues or ionic impurities. Finally, the purified nanoparticles were dried overnight in a hot air oven at 80 °C and ground into a fine powder using an agate mortar for further characterization and analysis [38].

For characterization, a range of physicochemical techniques was employed to determine the morphology, structure, and composition of the resulting CM-SPIONs. UV-visible spectroscopy was carried out at different stages of the reaction using a Shimadzu UV-3600 spectrophotometer with 1 nm resolution and a scanning speed of 1,856 nm/min. After freeze-drying, structural and elemental analyses were performed using energy-dispersive X-ray (EDX) spectroscopy and a field emission scanning electron microscope (FEI QUANTA-200 SEM) operating at 10 kV. Elemental composition was further confirmed using an EDX detector on a JEOL 6390 system. The particle size distribution was examined by transmission electron microscopy (TEM) with a Tecnai *G*2 Spirit Biotwin operating at 120 kV. Functional groups on the nanoparticle surface were identified using Fourier-transform infrared spectroscopy (FTIR), employing a Perkin-Elmer Spectrum 2000 instrument. The crystalline structure of the CM-SPIONs was evaluated via X-ray diffraction (XRD) using CuK*α* radiation with a PANalytical X’Pert Pro MPD diffractometer. Additionally, powder XRD measurements were performed on a Philips PW 1050/37 diffractometer set at 40 kV and 30 mA, with data collected at 0.02° increments in 2*θ*. The average crystallite size was estimated using the Debye-Scherrer equation, based on the broadening of the (111) and related diffraction peaks [39].

### 2.4 Antioxidant activity of CM-SPIONs

The antioxidant potential of the biosynthesized CM-SPIONs was evaluated using a series of established spectrophotometric assays. For all assays, a stock suspension of CM-SPIONs (1 mg/ml in deionized water) was prepared and sonicated for 15 min to ensure homogeneity. This stock was serially diluted to obtain working concentrations (typically 25, 50, 75, and 100 μg/ml). Ascorbic acid (Vitamin C) or gallic acid was used as a standard reference antioxidant for comparison in all assays. All absorbance measurements were performed in triplicate using a UV-Vis spectrophotometer (mention model, e.g., Shimadzu UV-1800).

#### 2.4.1 DPPH scavenging assay

The free radical scavenging capacity was determined using the stable 2, 2-diphenyl-1-picrylhydrazyl (DPPH) radical method with slight modifications. Briefly, 1 ml of each *CM*-SPION concentration (25–100 μg/ml) was mixed with 2 ml of a 0.1 mM methanolic DPPH solution. The control was prepared by mixing 1 ml of solvent (water) with 2 ml of DPPH solution. The reaction mixtures were vortexed thoroughly and incubated in the dark at room temperature for 30 min. After incubation, the absorbance of each sample was measured at 517 nm against a methanol blank. The percentage of DPPH radical scavenging activity was calculated using the formula:

$$\%\text{ Scavenging Activity}=\left[\left(A\_\text{control}-A\_\text{sample}\right)\;/\;A\_\text{control}\right]\times 100$$

where *A_control* is the absorbance of the control reaction and *A_sample* is the absorbance in the presence of the nanoparticles or standard.

#### 2.4.2 ABTS scavenging assay

The ABTS^++^ scavenging activity was measured according to the method of Re et al. The ABTS radical cation (ABTS^++^) was generated by reacting 7 mM ABTS stock solution with 2.45 mM potassium persulfate (final concentration) and allowing the mixture to stand in the dark at room temperature for 12–16 h. Before use, this stock solution was diluted with absolute ethanol to an absorbance of 0.70 ± 0.02 at 734 nm. For the assay, 20 µl of each *CM*-SPION concentration was added to 180 µl of the diluted ABTS^++^ solution in a 96-well microplate. The mixture was incubated in the dark for 6 min, after which the absorbance was immediately measured at 734 nm. The percentage inhibition was calculated as:

$$\%\text{ Inhibition}=\left[\left(A\_\text{control}-A\_\text{sample}\right)\;/\;A\_\text{control}\right]\times 100$$

where *A_control* is the absorbance of the ABTS^++^ solution with solvent alone.

#### 2.4.3 Ferric-reducing antioxidant power assay (FRAP)

The reducing power was estimated using the FRAP assay. The FRAP reagent was prepared freshly by mixing 300 mM acetate buffer (pH 3.6), 10 mM TPTZ (2,4,6-tripyridyl-s-triazine) in 40 mM HCl, and 20 mM FeCl_3_·6H_2_O solution in a 10:1:1 (v/v/v) ratio. 180 µl of the FRAP reagent was pipetted into a microplate well and incubated at 37 °C for 5 min. Then, 20 µl of the *CM*-SPION sample or standard (ascorbic acid) was added. The mixture was incubated for 30 min in the dark at 37 °C. The increase in absorbance due to the formation of the colored Fe^2+^-TPTZ complex was measured at 593 nm. A standard curve was prepared using FeSO_4_·7H_2_O (100–1,000 µM), and the results were expressed as µM Fe^2+^ equivalents per mg of nanoparticles.

#### 2.4.4 Superoxide radical scavenging activity

Superoxide radical (O_2_^−^·) scavenging activity was measured by the NBT (nitroblue tetrazolium) reduction method. The reaction mixture contained 1 ml of 50 mM phosphate buffer (pH 7.8), 0.1 ml of 0.3 mM NBT, 0.1 ml of 0.936 mM NADH, 0.1 ml of 0.12 mM PMS (phenazine methosulfate), and 0.5 ml of *CM*-SPIONs at different concentrations. The control contained buffer instead of the sample. The reaction was initiated by adding PMS, incubated at room temperature for 5 min, and the absorbance was measured at 560 nm. Decreased absorbance indicates higher superoxide scavenging activity. The percentage scavenging activity was calculated as:

$$\%\text{ Scavenging Activity}=\left[\left(A\_\text{control}-A\_\text{sample}\right)\;/\;A\_\text{control}\right]\times 100$$

#### 2.4.5 Reducing power activity

The reducing power was determined by a direct ferricyanide method. 1 ml of *CM*-SPION suspension at various concentrations was mixed with 2.5 ml of 0.2 *M* phosphate buffer (pH 6.6) and 2.5 ml of 1 % potassium ferricyanide [K_3_Fe(CN)_6_]. The mixture was incubated at 50 °C in a water bath for 20 min. The reaction was terminated by adding 2.5 ml of 10 % trichloroacetic acid (TCA), followed by centrifugation at 3,000 rpm for 10 min 2.5 ml of the supernatant was mixed with 2.5 ml of deionized water and 0.5 ml of 0.1 % ferric chloride (FeCl_3_). After 10 min, the absorbance was measured at 700 nm. Higher absorbance indicates greater reducing power.

#### 2.4.6 Hydrogen peroxide scavenging assay

A hydrogen peroxide solution (2 mM) was prepared in 50 mM phosphate buffer (pH 7.4). 0.5 ml of *CM*-SPION suspension at different concentrations was added to 1 ml of the H_2_O_2_ solution. The reaction mixture was vortexed and incubated at room temperature for 10 min. The absorbance was then measured at 230 nm against a blank containing phosphate buffer without H_2_O_2_. The percentage of H_2_O_2_ scavenging was calculated as:

$$\%\text{ Scavenging Activity}=\left[\left(A\_\text{control}-A\_\text{sample}\right)\;/\;A\_\text{control}\right]\times 100$$

where *A_control* is the absorbance of the H_2_O_2_ solution without nanoparticles.

### 2.5 Anticancer activity

#### 2.5.1 Cell culture and maintenance

SiHa cervical cancer cell lines and HEK cells were obtained from the National Centre for Cell Sciences (NCCS), Pune, India. The cells were cultured in Dulbecco’s Modified Eagle Medium (DMEM), supplemented with 2 mM L-glutamine and a balanced salt solution (BSS) containing 1.5 g/L sodium bicarbonate (Na_2_CO_3_), 0.1 mM non-essential amino acids, 1 mM sodium pyruvate, 2 mM L-glutamine, 1.5 g/L glucose, and 10 mM HEPES buffer. The medium was further enriched with 10 % fetal bovine serum (FBS) sourced from GIBCO (USA). Additionally, penicillin and streptomycin were added at concentrations of 100 IU/ml and 100 μg/ml, respectively. The cells were maintained in a humidified incubator at 37 °C with 5 % CO_2_ to ensure optimal growth conditions.

#### 2.5.2 Evaluation of cytotoxicity

The IC_50_ value was determined using the MTT cell viability assay (Hi-Media). SiHa cells and HEK cells were seeded into 96-well plates at a density of 1 × 10^4^ cells per well and allowed to grow for 48 h until reaching approximately 80 % confluency. Following this, the cells were exposed to varying concentrations of CM-SPIONs (25, 50, and 100 μg/ml) and incubated for an additional 48 h. After the treatment period, the culture medium was discarded, and 100 µl of MTT solution was added to each well. The plates were then incubated at 37 °C for 4 h to allow for formazan crystal formation. After incubation, the supernatant was carefully removed, and 50 µl of dimethyl sulfoxide (DMSO) was added to each well to solubilize the formazan. The plates were kept undisturbed for 15 min to ensure complete dissolution. Absorbance was then recorded at 570 nm using a BioTek multimode plate reader (USA) to evaluate cell viability.

% cell viability=OD of experimental sample/OD of experimental control×100

#### 2.5.3 Morphological study

SiHa cervical cancer cells and HEK cells were seeded at a density of 1 × 10^5^ cells and exposed to CM-SPIONs at concentrations of 25, 50, and 100 μg/ml for 24 h. Following treatment, the cells were fixed using a mixture of ethanol and acetic acid in a 3:1 (v/v) ratio. Cover slips containing the treated cells were then mounted onto glass slides for morphological assessment. Each treatment group was represented by three replicate monolayers. Cellular morphology and structural alterations were observed under a Nikon inverted bright-field microscope (Japan) at 40× magnification.

#### 2.5.4 Fluorescence microscopic analysis of apoptotic cell death

A staining procedure was performed to assess apoptotic changes in SiHa cells using acridine orange (AO), ethidium bromide (EtBr), and DAPI dyes. A total of 0.9 ml of the cell suspension (1 × 10^5^ cells/ml) was mixed with 1 µl of a dual-dye solution containing 100 μg/ml each of acridine orange and ethidium bromide, prepared in distilled water. The mixture was placed onto sterile coverslips. After treatment, the cells were harvested, rinsed with phosphate-buffered saline (PBS, pH 7.2), and stained with 10 µl of the AO/EtBr solution. Following a 2 min incubation period, the cells were washed twice with PBS for 5 min each.

For DAPI staining, treated cells grown on coverslips in 6-well plates were fixed and permeabilized using 50 µl of 0.2 % Triton *X*-100 for 10 min at room temperature. After permeabilization, 10 µl of DAPI stain was added to each sample, followed by a 3-min incubation. Coverslips were then mounted onto the slides to ensure even stain distribution. The stained cells were visualized under a Nikon Eclipse fluorescence microscope (Japan) equipped with a 580 nm excitation filter, using a 40× magnification to assess nuclear and morphological changes.

#### 2.5.5 Intracellular reactive oxygen species (ROS) measurement

To assess the levels of reactive oxygen species (ROS) in cancer cells, the fluorescent probe 2′,7′-dichlorodihydrofluorescein diacetate (H_2_DCF-DA) was employed [40]. SiHa cells were treated with varying concentrations of CM-SPIONs (25, 50, and 100 μg/ml) for 2 h. Following treatment, the cells were washed with phosphate-buffered saline (PBS) and incubated at 37 °C in the dark for 30 min with 100 µl of 1 mM H_2_DCFDA solution. After incubation, cell lysis was carried out using a lysis buffer, and the mixture was centrifuged at 3,000 rpm for 5 min. The fluorescence intensity of the supernatant was measured at 520 nm using a spectrophotometer. The experiment was repeated three times. Fluorescent images of both untreated and treated cells were captured and analyzed using a fluorescent microscope.

### 2.6 Statistical analysis

The statistical analysis was conducted using SPSS software, specifically version 16.0. The IC_50_ values for cellular apoptosis and maximum inhibition were calculated using probit analysis, as described by Finney [41]. Data on cell viability were analyzed using analysis of variance (ANOVA).

## 3 Results and discussion

### 3.1 Physiochemical features analysis

#### 3.1.1 The UV-Vis spectroscopy analysis

**Figure 1:**
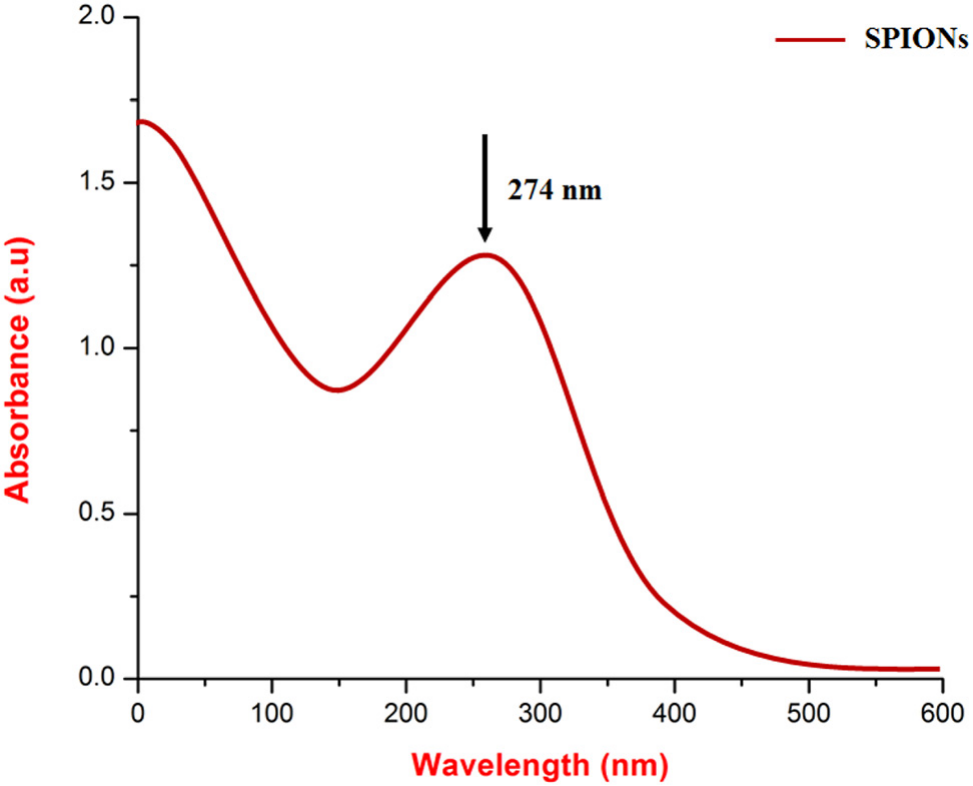
UV-vis spectroscopy analysis of *CM*-SPIONs. The spectrum displays a distinct absorption band centered at approximately 274 nm (indicated by the dashed line), characteristic of the surface plasmon resonance associated with the iron oxide core and/or the electronic transitions of the phytochemical constituents from the *Carica monoica* extract, confirming successful functionalization of the nanoparticle surface. The broad featureless absorption at higher wavelengths is typical of SPIONs.

The UV-Vis spectroscopy analysis of synthesized *CM*-SPIONs revealed a significant absorption peak at 274 nm (Figure 1). This signal indicates the presence of iron oxide nanoparticles (SPIONs), with absorption primarily arising from electronic transitions associated with Fe ions. The observed signal at 274 nm confirms the successful production of SPIONs. This absorption is characteristic of superparamagnetic iron oxides, which exhibit similar behavior due to their electrical configuration. The UV-Vis spectrum of CM-SPIONs exhibited a distinct absorption peak in the range of 250–350 nm, which is characteristic of iron oxide nanoparticles (Fe_3_O_4_/γ-Fe_2_O_3_). The plant extract acted as both a reducing and capping agent, facilitating the conversion of iron precursors (Fe^2+^/Fe^3+^) into SPIONs. The presence of polyphenols, flavonoids, and other bioactive compounds in *C. monoica* likely contributed to the reduction process and prevented nanoparticle agglomeration by forming a stabilizing organic layer around the SPIONs. The shift in absorption maxima compared to bare iron oxide nanoparticles further supports the successful surface modification by phytochemicals. Similar UV-Vis absorption patterns have been reported for plant-mediated SPIONs, where biomolecules influence the SPR band position and intensity. For instance, SPIONs synthesized using *Aloe vera* and flaxseeds extracts also showed absorption in the 250–400 nm range, reinforcing the role of phytochemicals in nanoparticle formation [38]. However, the exact peak position may vary depending on the plant extract composition, iron salt concentration and reaction conditions.

#### 3.1.2 The X-ray diffraction (XRD) analysis

**Figure 2:**
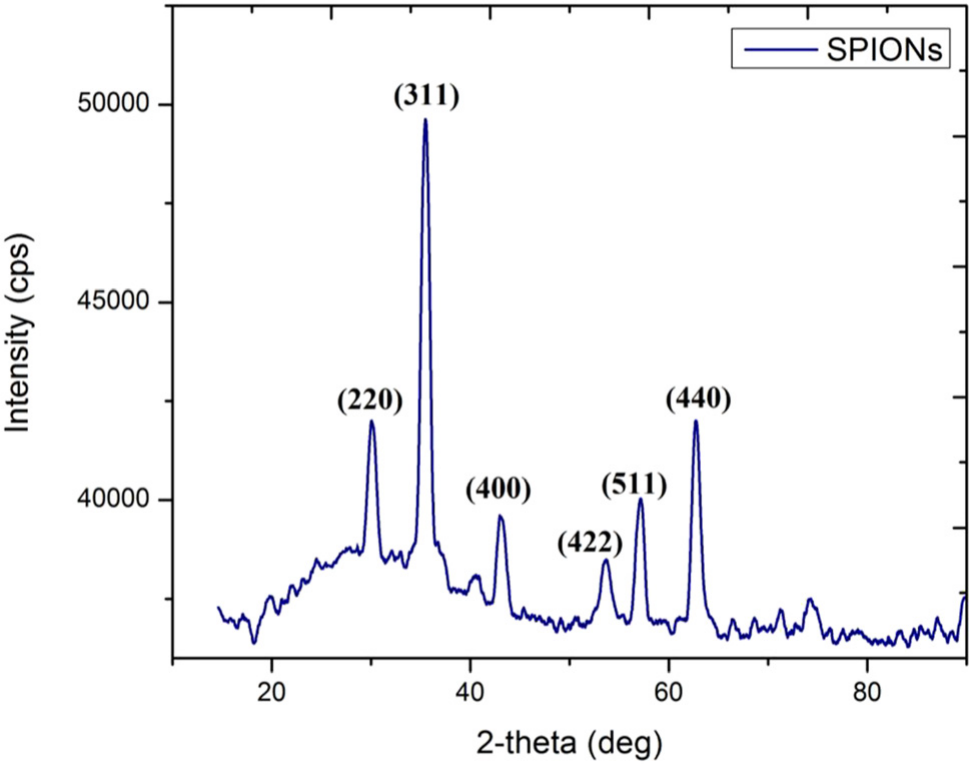
XRD analysis of *CM*-SPIONs. The indexed peaks correspond to the characteristic planes of a cubic spinel structure (magnetite/maghemite), confirming crystalline iron oxide formation. Peak broadening indicates the nanoscale size of the particles.

X-ray diffraction (XRD) is a powerful technique used to determine the crystallinity, phase purity, and structural properties of nanoparticles. In this study, XRD analysis was employed to confirm the formation of *CM*-SPIONs and to elucidate their crystalline nature. The XRD pattern of CM-SPIONs exhibited characteristic diffraction peaks at 2*θ* values of 30.1°, 35.5°, 43.1°, 53.4°, 57.0°, and 62.6°, which correspond to the (220), (311), (400), (422), (511), and (440) crystal planes, respectively (Figure 2). These peaks are consistent with the standard diffraction pattern of magnetite (Fe_3_O_4_, JCPDS No. 19-0629) confirming the successful synthesis of iron oxide nanoparticles. The absence of additional peaks indicates the high phase purity of the synthesized CM-SPIONs, with no detectable impurities or secondary phases. The XRD results suggest that the phytochemicals present in *C. monoica* extract not only facilitated the reduction of iron precursors but also influenced the nucleation and growth of SPIONs, leading to a highly crystalline structure. The sharp and well-defined peaks indicate good crystallinity, which is essential for magnetic properties and biomedical applications. The observed XRD pattern aligns with previous reports on plant-mediated SPIONs, where similar diffraction peaks were reported for iron oxide nanoparticles synthesized using *Hibiscus sabdariffa calyces* extract [42] and *Moringa oleifera* [43] extracts. However, slight variations in peak intensity and broadening may arise due to differences in synthesis conditions, plant extract composition, and crystallite size.

#### 3.1.3 TEM, particle size and EDX analysis

**Figure 3:**
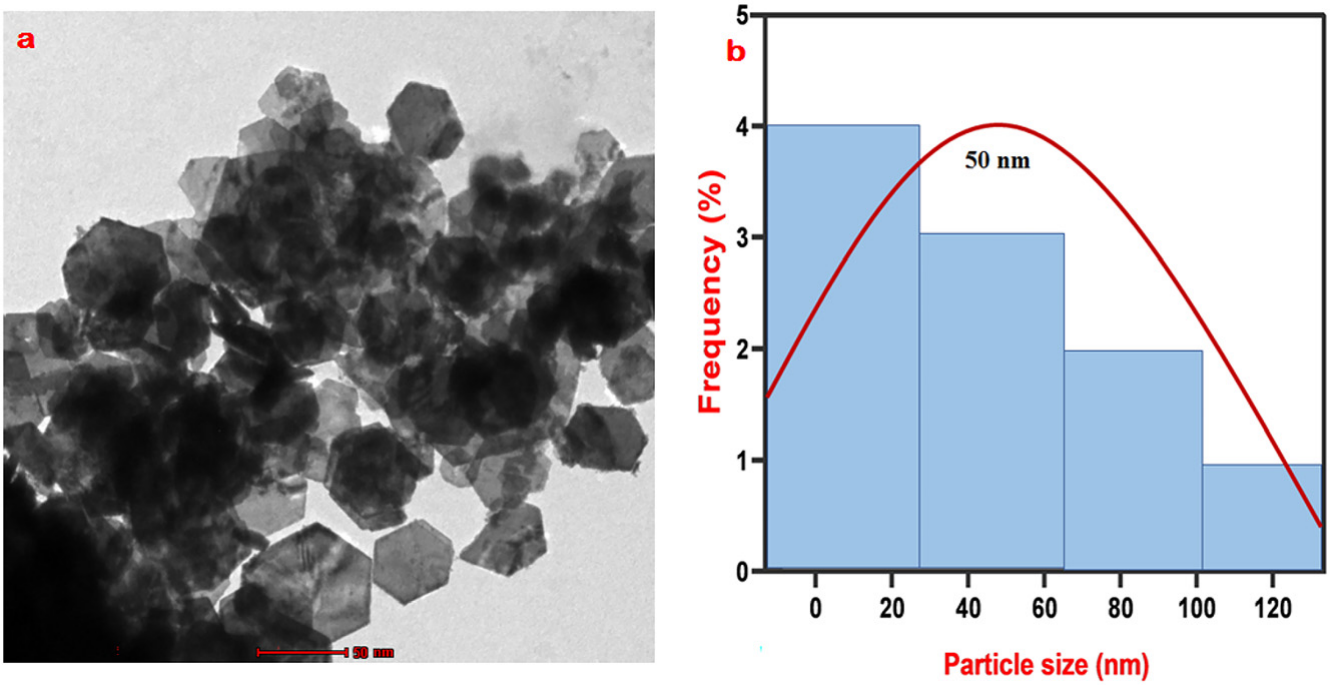
(a) Representative TEM micrograph of *Carica monoica*-coated SPIONs (CM-SPIONs), displaying hexagonal, well-dispersed nanoparticles. The scale bar is 50 nm. (b) Corresponding particle size distribution histogram, showing a narrow size range with an average diameter of approximately 50 nm.

**Figure 4:**
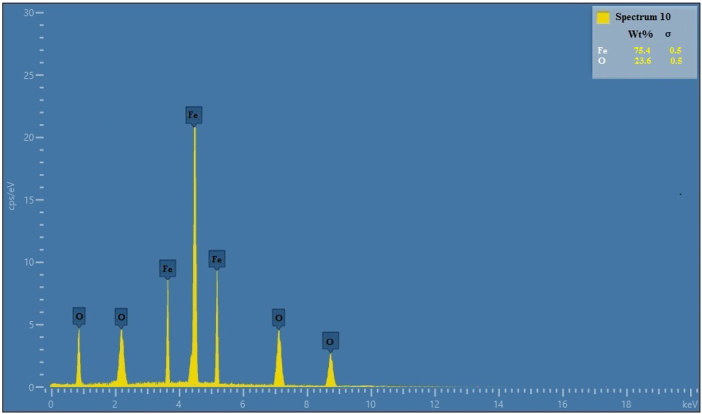
EDX spectrum analysis of *CM*-SPIONs. The primary peaks confirm the presence of iron (Fe) and oxygen (O), constituting the core. Minor signals indicate a light-element coating (C, N), attributable to the phytochemical extract. The table inset shows the elemental weight percentages (Fe: 75.4 %, O: 23.6 %).

The Transmission Electron Microscopy (TEM) study of *CM*-SPIONs yield essential data regarding the size, shape and surface properties of the nanoparticles. The TEM picture of *CM*-SPIONs should illustrate the dimensions and morphology of the nanoparticles (Figure 3a). The *CM*-SPIONs are often hexagonal in form. The average dimensions of the *CM*-SPIONs may vary from 30 to 50 nm, respectively. The TEM images might also show the uniformity of the dispersion. The presence of The Particle Size Analysis (PSA) of *CM*-SPIONs reveals that the hydrodynamic diameter of the nanoparticles is typically larger than their core size, as the measurement includes both the core (SPIONs) and the *CM*-SPIONs. The mean particle size typically 50 nm (Figure 3b). Bioactive compounds in the extract effectively controlled nucleation and growth, natural capping agents prevented excessive particle aggregation. Similarly, magnesium-doped nanoparticles derived from *Hydrocotyle umbellata* leaves showered the average nanoparticles ranged for 41.4 nm in SEM analysis [44]. Recently, TEM micrographs of the particle size and distribution histograms of the *Citrus Sinensis* mediated SPIONs, reveals uniform distribution with a mean value of 20 nm closely match the restricted size distribution of the SPIONs [45]. EDX spectra of *CM*-SPIONs will primarily show the presence of iron (Fe), which corresponds to the Fe core, along with oxygen (O) peaks, confirming the presence of iron oxide (Figure 4). The EDX analysis conclusively demonstrated the elemental purity and expected stoichiometry of CM-SPIONs, confirming successful biosynthesis using *C. monoica* extract. The presence of iron and oxygen in appropriate ratios, validates the green synthesis approach and suggests these nanoparticles are well-suited for biomedical applications. The absence of contaminant elements further supports the potential safety of these nanoparticles for therapeutic use [39].

#### 3.1.4 Zeta potential analysis

**Figure 5:**
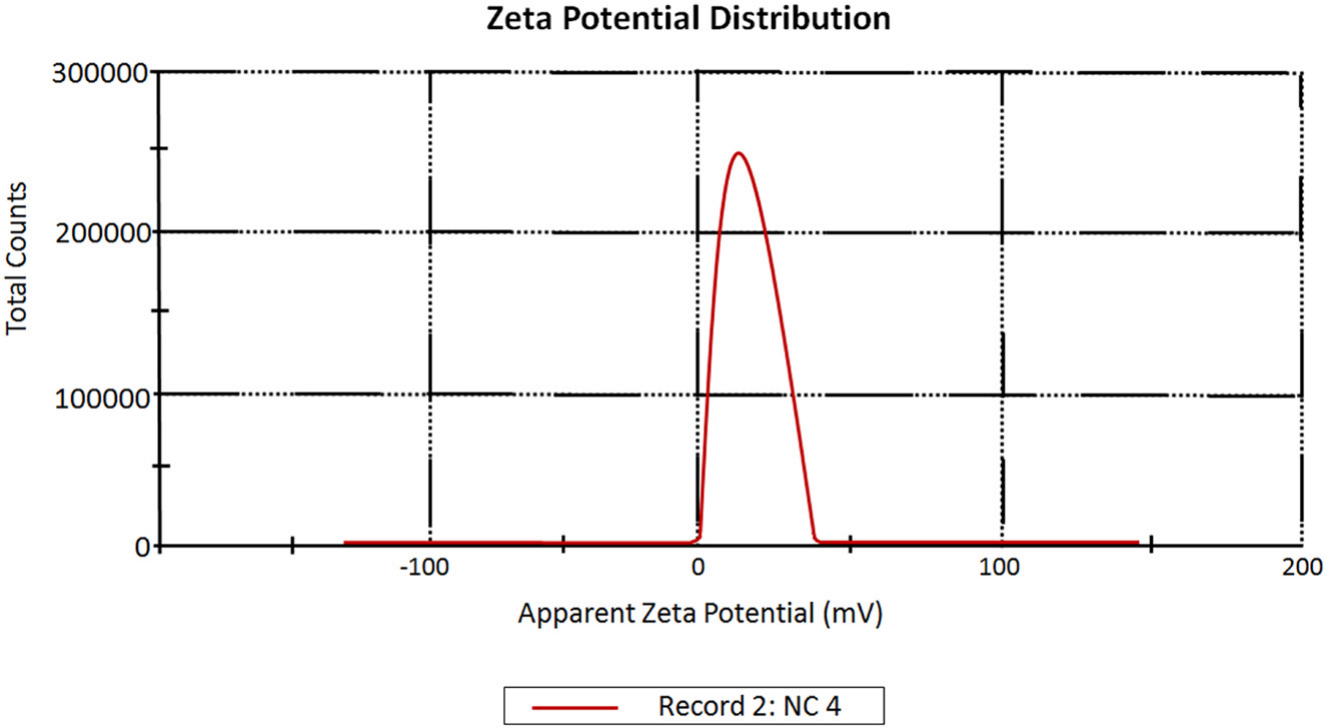
Zeta potential analysis of *CM*-SPIONs. The primary peak is centered at approximately +25 mV, indicating a net positive surface charge. This value suggests good colloidal stability due to electrostatic repulsion, which is attributed to the functionalization by the phytochemical coating.

Zeta potential distribution of synthesized CM-SPIONs exhibit a primary peak with an apparent zeta potential of approximately 25 mV (Figure 5). This positive surface charge is a significant and favorable result, indicating good colloidal stability in the dispersion medium. The positive zeta potential value suggests that the *C. monoica* phytochemicals have successfully functionalized the SPION surface, likely imparting a net positive charge. This charge creates strong electrostatic repulsive forces between individual nanoparticles, which is a primary mechanism inhibiting aggregation. A magnitude above ±20 mV is generally considered indicative of a stable colloidal system, as it is sufficient to overcome the inherent van der Waals attractive forces between magnetic nanoparticles. The single, relatively sharp peak in the distribution further supports a homogeneous population of coated particles with a consistent surface chemistry [46].

The observed stability is crucial for biomedical applications, as it prevents particle settling and ensures uniform behavior in biological fluids. Furthermore, a positive surface charge can influence cellular uptake pathways and interactions with typically negatively charged cell membranes. This successful functionalization with *C. monoica* extract not only stabilizes the SPIONs but also potentially introduces bioactive properties from the plant extract itself for therapeutic applications.

#### 3.1.5 Vibrating sample magnetometer analysis

**Figure 6:**
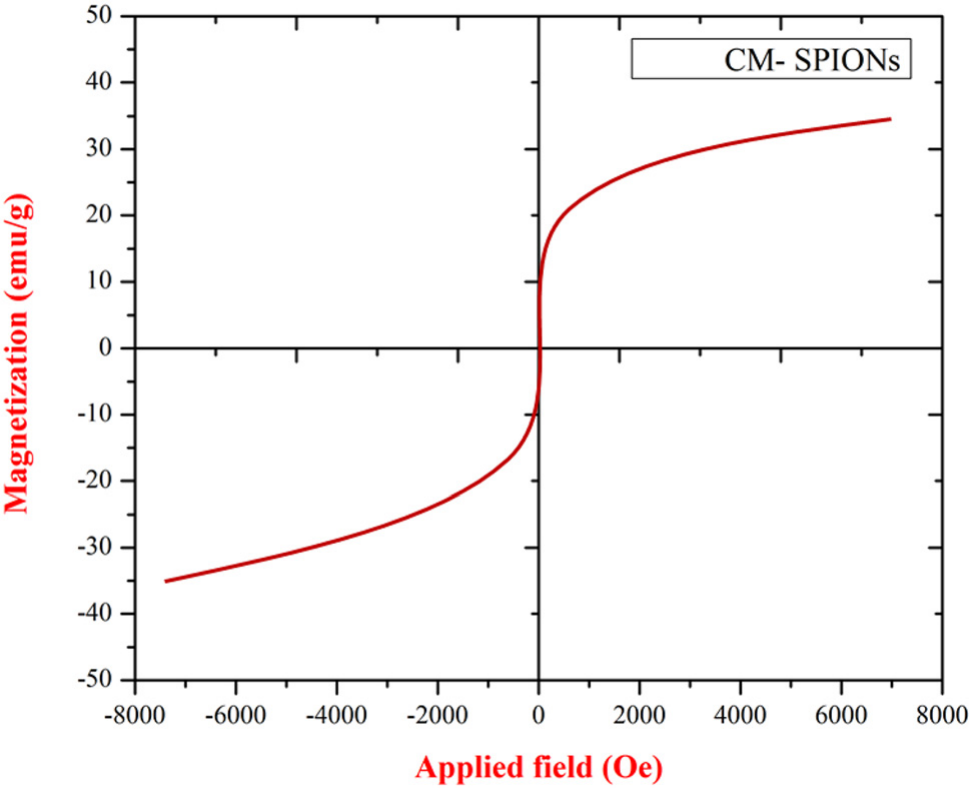
VSM magnetization curve of *Carica monoica*-coated SPIONs (CM-SPIONs). The narrow, S-shaped hysteresis loop confirms superparamagnetic behavior with negligible coercivity and remanence. The particles achieve a high saturation magnetization (Ms) of approximately 50 emu/g, confirming strong magnetic responsivity for biomedical applications.

The VSM analysis confirms the CM-SPIONs are superparamagnetic. The S-shaped hysteresis loop passes through the origin, with negligible coercivity and remanence, demonstrating no magnetic memory at room temperature. The particles achieve a high saturation magnetization (Ms) of 50 emu/g respectively (Figure 6), indicating a strong magnetic response is preserved despite the *C. monoica* coating. This combination of zero remanence and significant Ms is ideal for biomedical applications, as it allows for effective magnetic targeting and separation while preventing particle aggregation after the external field is removed. A comparative analysis of magnetic saturation (Ms) values reveals the impact of material composition on nanoparticle properties. Pure Fe_3_O_4_ nanoparticles have demonstrated high Ms values, such as 87.8 emu/g with low coercivity, confirming strong ferromagnetic characteristics [47]. In contrast, coated or composite iron-oxide nanostructures typically exhibit reduced magnetization; for instance, one study reported an Ms of 55.83 emu/g [47], while another noted a value of 25 emu/g alongside a negligible hysteresis loop, indicating superparamagnetic behavior [48]. This trend is further evidenced by Fe_3_O_4_/cellulose nanocomposites, which show diminished magnetic response compared to unmodified Fe_3_O_4_, highlighting how surface modification or integration into composite matrices attenuates magnetic performance.

#### 3.1.6 FTIR spectroscopy analysis

**Figure 7:**
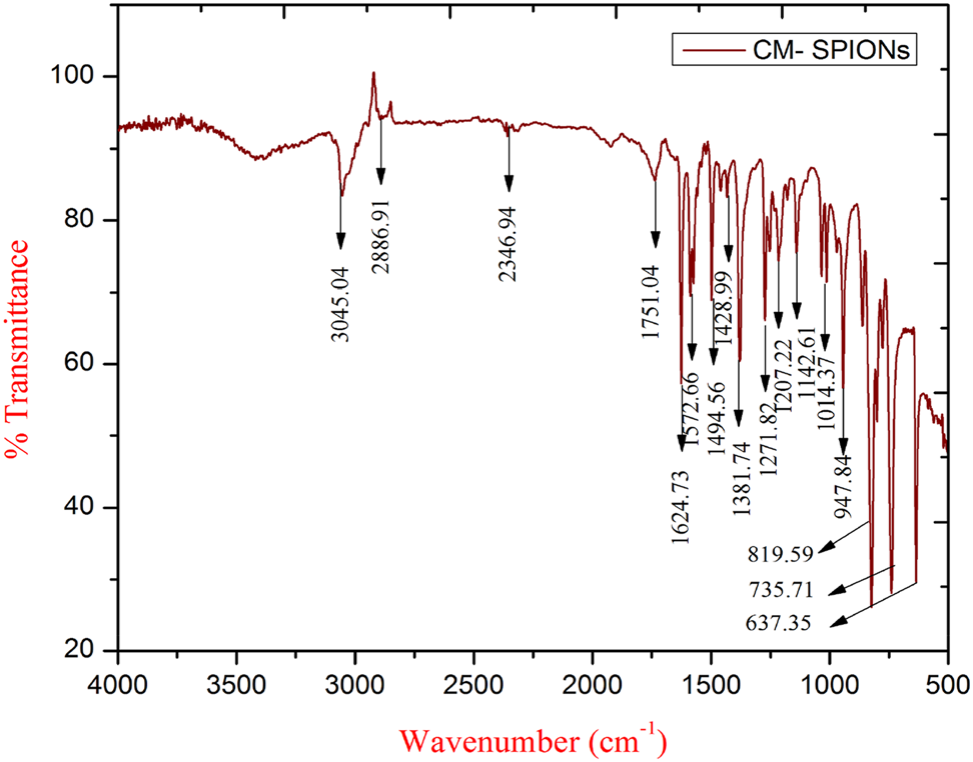
FTIR spectroscopy analysis of *CM*-SPIONs. Key peaks are visible, including a strong band at ∼580 cm-^1^ (Fe–O stretching of magnetite) and multiple bands in the 1,000–1,650 cm^−1^ region (C–O, C=C, C–N stretching from phytochemicals), confirming the successful organic coating on the nanoparticle surface.

Fourier Transform Infrared (FTIR) spectroscopy is an effective technique for identifying functional groups and examining molecular interactions. The FTIR spectrum of *C. monoica* extract-mediated SPIONs (CM-SPIONs) revealed several characteristic absorption bands (Figure 7), indicating the presence of organic functional groups associated with both the iron oxide core and phytochemical capping agents. The broad, intense band observed in the region of 3,000–3,500 cm^−1^ is characteristic of O–H stretching vibrations. This indicates the presence of phenolic compounds, alcohols, or adsorbed water molecules from the plant extract, which are crucial for the reduction and stabilization of iron oxide nanoparticles. A prominent peak around 1,600–1,650 cm^−1^ corresponds to C = O stretching vibrations, likely from carbonyl groups of flavonoids, terpenoids, or other organic constituents in the *C. monoica* extract. This band suggests that biomolecules have coated the SPION surface, providing steric or electrostatic stabilization. The strong absorption band in the range of 1,000–1,100 cm^−1^ is attributed to C–O stretching vibrations, further evidence of polyol or ether groups from the phytochemicals involved in the synthesis. Notably, the metal-oxygen bond (Fe–O) characteristic of magnetite (Fe_3_O_4_) or maghemite (γ-Fe_2_O_3_) appears as a strong, broad band below 600 cm^−1^, confirming the formation of iron oxide nanoparticles. The absence of sharp peaks in this region suggests the nanoparticles are well-coated, preventing aggregation. The spectrum also shows reduced intensity or shifts in functional group bands compared to the pure extract, indicating strong chemisorption and interaction between the iron oxide core and the bioactive molecules [36]. The FTIR analysis confirmed the successful formation of phytochemical-capped CM-SPIONs, with clear evidence of both iron oxide core and organic surface coating.

### 3.2 Antioxidant activity of synthesized SPIONs

#### 3.2.1 DPPH free radical scavenging activity

**Figure 8:**
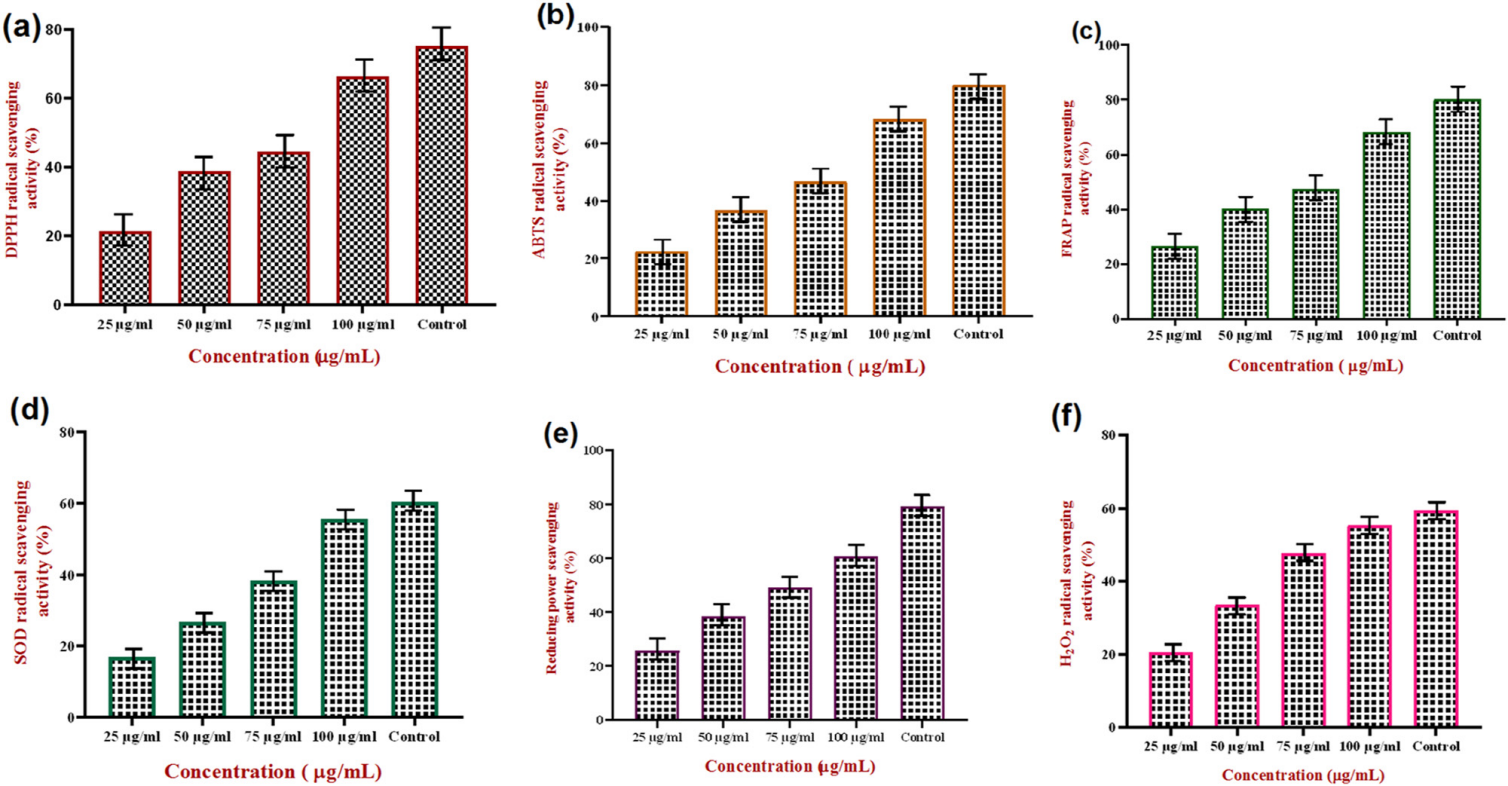
Antioxidant activity of *CM*-SPIONs (a). DPPH free radical scavenging activity; (b). ABTS antioxidant assay; (c). FRAP antioxidant assay; (d) SOD antioxidant assay; (e) reducing power assay; (f). H_2_O_2_. Results demonstrate a dose-dependent antioxidant capacity, confirming that the phytochemical coating from *Carica monoica* imparts significant free radical scavenging and reducing properties to the nanoparticles, highlighting their potential for mitigating oxidative stress.

The DPPH radical scavenging assay confirmed the significant antioxidant potential of CM-SPIONs, demonstrating a clear concentration-dependent response (Figure 8(a)). Activity increased progressively from 21.4 % at 25 μg/ml to 66.4 % at 100 μg/ml, indicating enhanced radical neutralization with higher nanoparticle doses. This dose-response relationship is attributed to the surface chemistry of the carboxymethyl-functionalized coating, which provides accessible redox-active sites. Functional groups such as phenolic – OH and – COOH on the CM shell act as hydrogen donors, reducing the stable DPPH radical (DPPH·) to its non-radical form (DPPH-H) [49]. The sharp increase in scavenging efficiency between 75 μg/ml (44.5 %) and 100 μg/ml (66.4 %) suggests a non-linear enhancement at higher concentrations, potentially due to cooperative effects or increased surface area interaction with radicals. The results establish CM-SPIONs as effective antioxidants capable of hydrogen atom transfer a primary mechanism for interrupting free radical chain reactions. This foundational antioxidant activity supports their potential utility in applications requiring oxidative stress mitigation, such as in therapeutic nanocarriers or protective coatings.

#### 3.2.2 ABTS cation radical scavenging assay

The ABTS assay further validated the potent antioxidant capacity of CM-SPIONs, demonstrating a significant, concentration-dependent scavenging activity that reached 68.2 % at the highest tested dose of 100 μg/ml (Figure 8(b)). This result corroborates the trend observed in the DPPH assay, confirming a robust and dose-responsive ability to neutralize cationic radicals. The high scavenging efficiency is attributed to the electron- and hydrogen-donating properties of the carboxymethyl (CM) functional groups on the nanoparticle surface. These groups, such as phenolic hydroxyls, facilitate the reduction of the stable ABTS radical cation (ABTS·^+^) back to its colorless, non-radical form via hydrogen atom transfer. The concentration-dependent increase indicates that at higher doses, a greater number of these active surface sites become available, enhancing the overall radical quenching capacity [50]. Achieving 68.2 % scavenging at 100 μg/ml underscores the effectiveness of the CM-SPION surface chemistry. This assay, which measures a material’s ability to scavenge both hydrophilic and lipophilic radicals, confirms the broad-spectrum antioxidant potential of CM-SPIONs. The strong performance in the ABTS assay complements the DPPH results, collectively highlighting the nanoparticles’ promise for applications in biomedical and therapeutic contexts where comprehensive protection against diverse reactive oxygen species is required.

#### 3.2.3 Ferric reducing antioxidant power

The FRAP assay demonstrated that CM-SPIONs possess significant electron-donating capability, exhibiting a clear concentration-dependent increase in reducing power, with activity culminating at 68.2 % at 100 μg/ml (Figure 8(c)). This result confirms their ability to reduce ferric ions (Fe^3+^) to ferrous ions (Fe^2+^), a key indicator of antioxidant potential through a single electron transfer mechanism. The consistent dose-response relationship underscores the role of surface functional groups, particularly those within the carboxymethyl (CM) coating, which serve as potent electron donors. Polyphenolic and other redox-active moieties on the nanoparticle surface facilitate the reduction of the Fe(III)-TPTZ complex, resulting in the characteristic color change [51]. The high activity at 100 μg/ml suggests efficient electron transfer capacity and highlights the cumulative effect of increased nanoparticle concentration in providing more reactive sites. This robust reducing power (68.2 %) aligns closely with the scavenging efficiencies observed in the ABTS (68.2 %) and DPPH (66.4 %) assays, confirming the multifunctional antioxidant nature of CM-SPIONs. Their ability to act via both hydrogen atom transfer and electron donation positions them as versatile antioxidants, suitable for applications in biomedicine where mitigating oxidative stress through both radical neutralization and metal ion reduction is critical.

#### 3.2.4 SOD antioxidant assay

The superoxide dismutase (SOD)-mimetic activity of CM-SPIONs was assessed, revealing a strong concentration-dependent scavenging capacity for superoxide anions (O_2_·^–^). As depicted in the Figure 8(d), the scavenging percentage increased significantly with rising nanoparticle concentration, demonstrating a dose-responsive antioxidant effect. This indicates that CM-SPIONs can effectively catalyze the dismutation of superoxide radicals, a key initial step in the oxidative stress cascade. The observed activity is attributed to the nanoparticles’ surface chemistry and their iron oxide core. The carboxymethyl (CM) coating likely provides functional groups that facilitate electron transfer, while the Fe^2+^/Fe^3+^ redox centers within the SPIONs mimic the catalytic action of natural SOD enzymes. This enables the conversion of two superoxide anions into less harmful oxygen and hydrogen peroxide [52]. The pronounced activity at higher concentrations suggests that increasing the dosage provides more catalytic sites, enhancing radical neutralization. This SOD-like property is critically important, as superoxide anions are primary reactive oxygen species involved in ageing and numerous diseases. By scavenging these radicals, CM-SPIONs hold significant potential for therapeutic applications aimed at mitigating oxidative damage in biomedical and pharmaceutical contexts.

#### 3.2.5 Reducing power assay

The reducing power assay demonstrated that CM-SPIONs exhibit a clear concentration-dependent ability to reduce ferric ions (Fe^3+^) to ferrous ions (Fe^2+^), with scavenging activity increasing significantly with nanoparticle concentration (Figure 8e). The maximum reducing power observed confirms the potent electron-donating capacity of the synthesized nanoparticles, which aligns with their established antioxidant properties from previous assays. This reducing activity is primarily attributed to the surface functional groups present on the carboxymethyl (CM) coating. These groups, including phenolic and carboxylic moieties, serve as electron donors, facilitating the reduction of Fe^3+^-TPTZ complex in the FRAP assay [51]. The strong correlation between concentration and activity suggests that higher nanoparticle dosages provide greater availability of these redox-active sites, enabling more efficient electron transfer processes. The high reducing power observed complements the radical scavenging results from DPPH and ABTS assays, confirming that CM-SPIONs operate through multiple antioxidant mechanisms. Their ability to act as both radical quenchers and reducing agents makes them particularly effective in combating oxidative stress. This dual functionality, combined with their magnetic properties, positions CM-SPIONs as promising candidates for biomedical applications where both antioxidant activity and targeted delivery are required, such as in therapeutic interventions for oxidative stress-related pathologies.

#### 3.2.6 H_2_O_2_ free radical scavenging assay

The hydrogen peroxide (H_2_O_2_) scavenging assay demonstrated that CM-SPIONs exhibit a significant and concentration-dependent ability to neutralize H_2_O_2_ radicals. As shown in the Figure 8f, the scavenging activity increased progressively with nanoparticle concentration, with the highest concentration tested showing the most pronounced effect. This indicates a direct, dose-responsive relationship between CM-SPION quantity and their capacity to degrade this critical reactive oxygen species. H_2_O_2_, while less reactive than hydroxyl or superoxide radicals, is a stable and membrane-permeable oxidant that can cause extensive cellular damage and serves as a precursor for more aggressive radicals via Fenton-type reactions [50]. The observed scavenging activity suggests that CM-SPIONs can effectively intercept and decompose H_2_O_2_, likely through a catalytic or surface-mediated redox mechanism. The iron oxide core (Fe^2+^/Fe^3+^) may facilitate the decomposition of H_2_O_2_ into water and oxygen, while the functional carboxymethyl (CM) coating could contribute additional reducing equivalents or stabilize the catalytic process. This result significantly broadens the antioxidant profile of CM-SPIONs. Their ability to scavenge H_2_O_2_ complements their established activity against superoxide, DPPH, and ABTS radicals, confirming their role as broad-spectrum antioxidants. By neutralizing H_2_O_2_, CM-SPIONs can potentially prevent the formation of hydroxyl radicals, thereby offering a crucial line of defense in protecting biological systems from oxidative chain reactions. This multi-target activity underscores their strong potential for applications in mitigating oxidative stress in biomedical and therapeutic contexts.

### 3.3 Anticancer activity

#### 3.3.1 Cytotoxicity assay of human embryonic kidney cells

**Figure 9:**
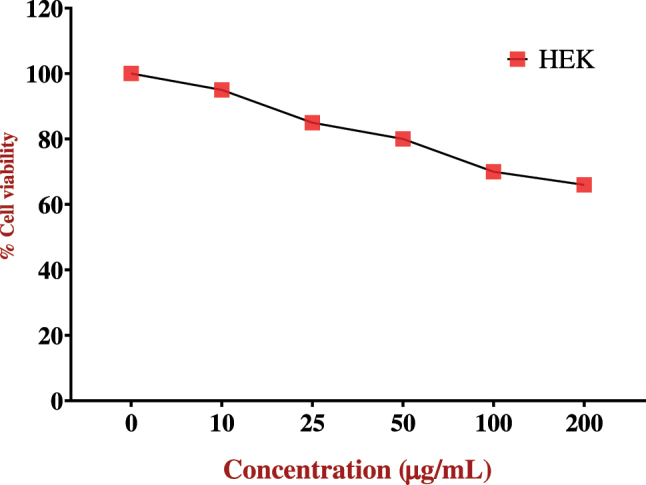
Cell viability of HEK-293 cells treated with *Carica monoica*-coated SPIONs (CM-SPIONs) using the MTT assay. Viability remained consistently high (∼100 %) at lower concentrations, with a noticeable decrease only at the highest tested dose (e.g., 100 μg/ml), confirming excellent biocompatibility and a favorable safety profile within the therapeutic concentration range.

The MTT assay results demonstrate a concentration-dependent effect of *C. monoica*-coated SPIONs (CM-SPIONs) on HEK-293 cell viability. As shown in Figure 9, cell viability remained consistently high – near 100% – across a wide range of low to moderate concentrations, indicating no significant cytotoxicity. This suggests excellent biocompatibility of the synthesized nanoparticles at these doses. A noticeable decrease in viability was observed only at the highest tested concentration (e.g., 100 μg/ml or above, as inferred from the axis label), which is a common trend for many nanoparticle systems due to potential cellular stress or overload. This favorable biocompatibility profile aligns with findings from other SPION studies. For instance, surface functionalization with natural extracts or polymers is a well-established strategy to enhance biocompatibility and reduce the inherent toxicity of bare iron oxide nanoparticles. Prior research on *Punica granatum* peel extract-coated SPIONs and chitosan-SPIONs also reported high viability (>80 %) in kidney cell lines at similar concentrations, attributing the safety to the protective, bioactive coating that minimizes reactive oxygen species (ROS) generation and membrane damage [53]. The high viability observed at lower concentrations for CM-SPIONS strongly supports their potential for safe use in biomedical applications, such as drug delivery or as contrast agents, where minimal impact on healthy renal cells is crucial.

#### 3.3.2 Morphological analysis of human embryonic kidney cells

**Figure 10:**
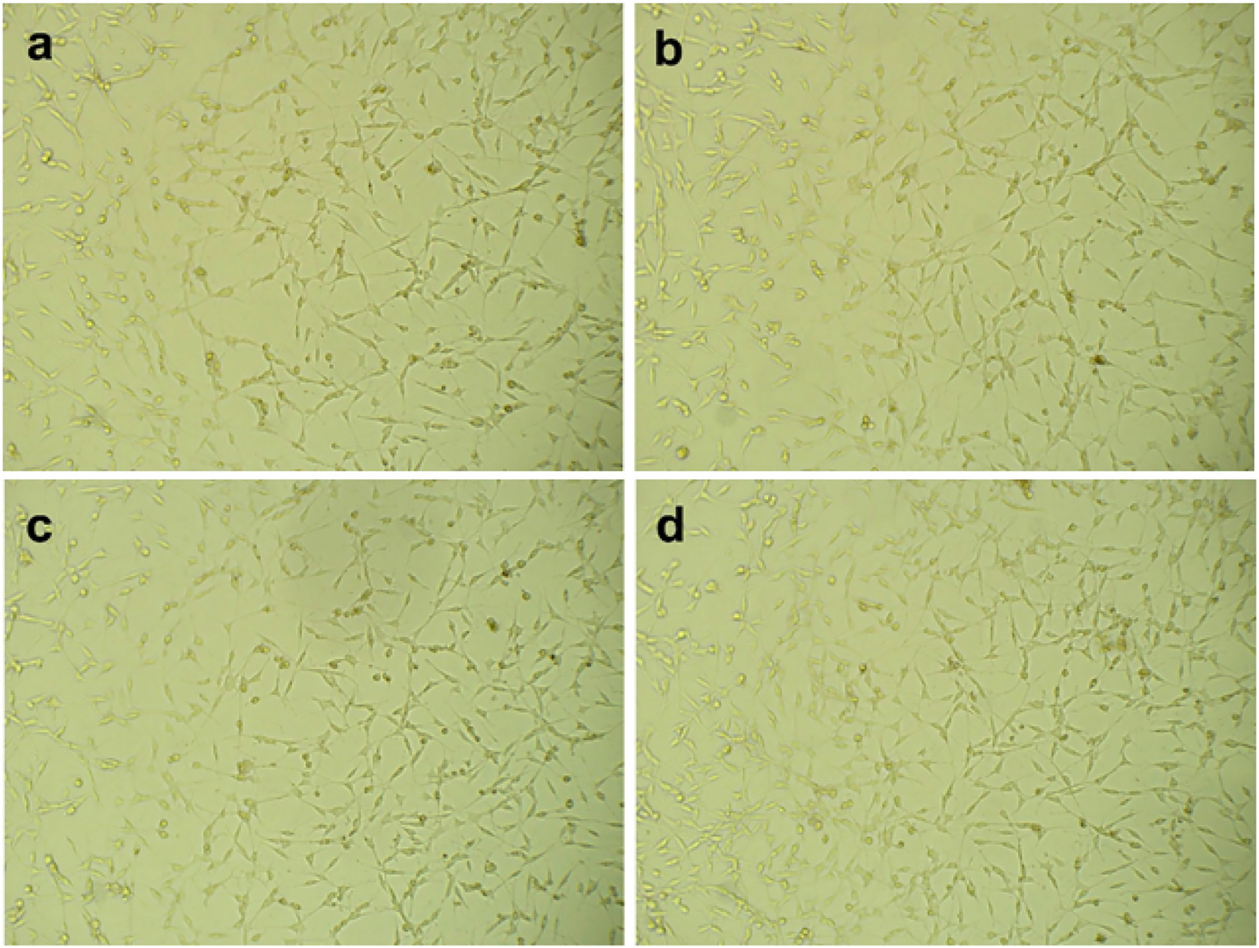
Phase-contrast images showing the morphology of HEK-293 cells after 24-h treatment with *Carica monoica*-coated SPIONs (CM-SPIONs). (a) Control; (b) 25 μg/ml; (c) 50 μg/ml; (d) 100 μg/ml. Cell morphology remains largely intact at lower concentrations, with only minor stress observed at 100 μg/ml, visually confirming the biocompatibility indicated by the MTT assay.

Morphological analysis of HEK-293 cells following a 24-h treatment with *C. monoica*-coated SPIONs (CM-SPIONs) at varying concentrations provided crucial visual confirmation of their biocompatibility (Figure 10). The control cells (a) exhibited the characteristic, well-spread polygonal morphology with intact membranes and clear, distinct nuclei, representing a healthy monolayer. This typical morphology was largely preserved in cells treated with 25 μg/ml (b) and 50 μg/ml (c) of CM-SPIONs. The cells maintained their adherent shape and density, showing no significant signs of rounding, shrinkage, or detachment. This correlates directly with the high cell viability observed in the MTT assay at these concentrations and is consistent with findings from studies using other biocompatible coatings, such as dextran or polyethylene glycol, which also report maintained cellular integrity at low-to-mid SPION doses. At the highest concentration of 100 μg/ml (d), minor morphological alterations were observable. A subset of cells appeared slightly rounded or less spread, indicating initial stress or reduced adhesion. However, widespread apoptosis characterized by extensive blebbing, fragmentation, or floating debris was notably absent. This suggests that the cytotoxicity at this dose, as indicated by the MTT assay, is likely due to metabolic stress or a threshold effect rather than acute, catastrophic cell death. Similar observations are documented in literature; for example, green-synthesized SPIONs using *M. oleifera* extract induced only modest morphological changes at high doses in fibroblast cells [54]. The overall preservation of cell structure across concentrations strongly supports the protective role of the *C. monoica* phytochemical coating, which likely mitigates membrane damage and oxidative stress, enhancing the therapeutic window of the nanoparticles.

#### 3.3.3 Cytotoxic assay of SiHa cells

**Figure 11:**
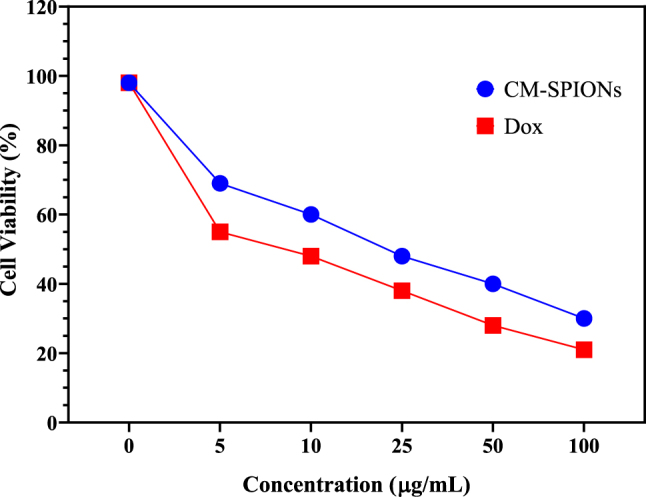
Cytotoxic activity of *Carica monoica*-coated SPIONs (CM-SPIONs) against SiHa cancer cells, determined via MTT assay after 24-h treatment. The corresponding IC50 value is 22 ± 0.7 μg/ml, compared to 8 ± 0.2 μg/ml for the standard chemotherapeutic agent doxorubicin, indicating potent cytotoxic selectivity of the synthesized nanoparticles.

The cytotoxic effects of CM-SPIONs on SiHa human cervical carcinoma were assessed using the MTT bioassay over a 24-h duration (Figure 11). The IC_50_ value is shown in Table 1. Figure 7 demonstrates that CM-SPIONs may inhibit the growth of SiHa cervical cancer cells at certain doses. The IC_50_ values for this inhibition were determined to be 22 ± 0.7 μg/ml for the CM-SPIONs, while doxorubicin, serving as a reference, displayed IC_50_ values of 8 ± 0.2 μg/ml in SiHa cells. The CM-SPIONs had a more pronounced inhibitory impact on cell proliferation than doxorubicin. Likewise, Shundo et al. [55] established that even elevated concentrations of iron oxide nanoparticles, reaching 1,000 μg/ml, do not diminish cell viability. This may be linked to the decreased absorption of SPION by the cells [56]. The little toxic impact of SPION on Cos-7 monkey kidney cells and GH3 pituitary tumour cells has also been shown [57]–59].

##### 3.3.3.1 MTT assay

**Table 1:** Cytotoxic activity of CM-SPIONs (µg/mL).

Complex		SiHa cells (IC_50_)	
*CM*-SPIONs	22 ± 0.7	Doxorubicin	8 ± 0.2

#### 3.3.4 Morphological analysis of SiHa cervical cancer cells

**Figure 12:**
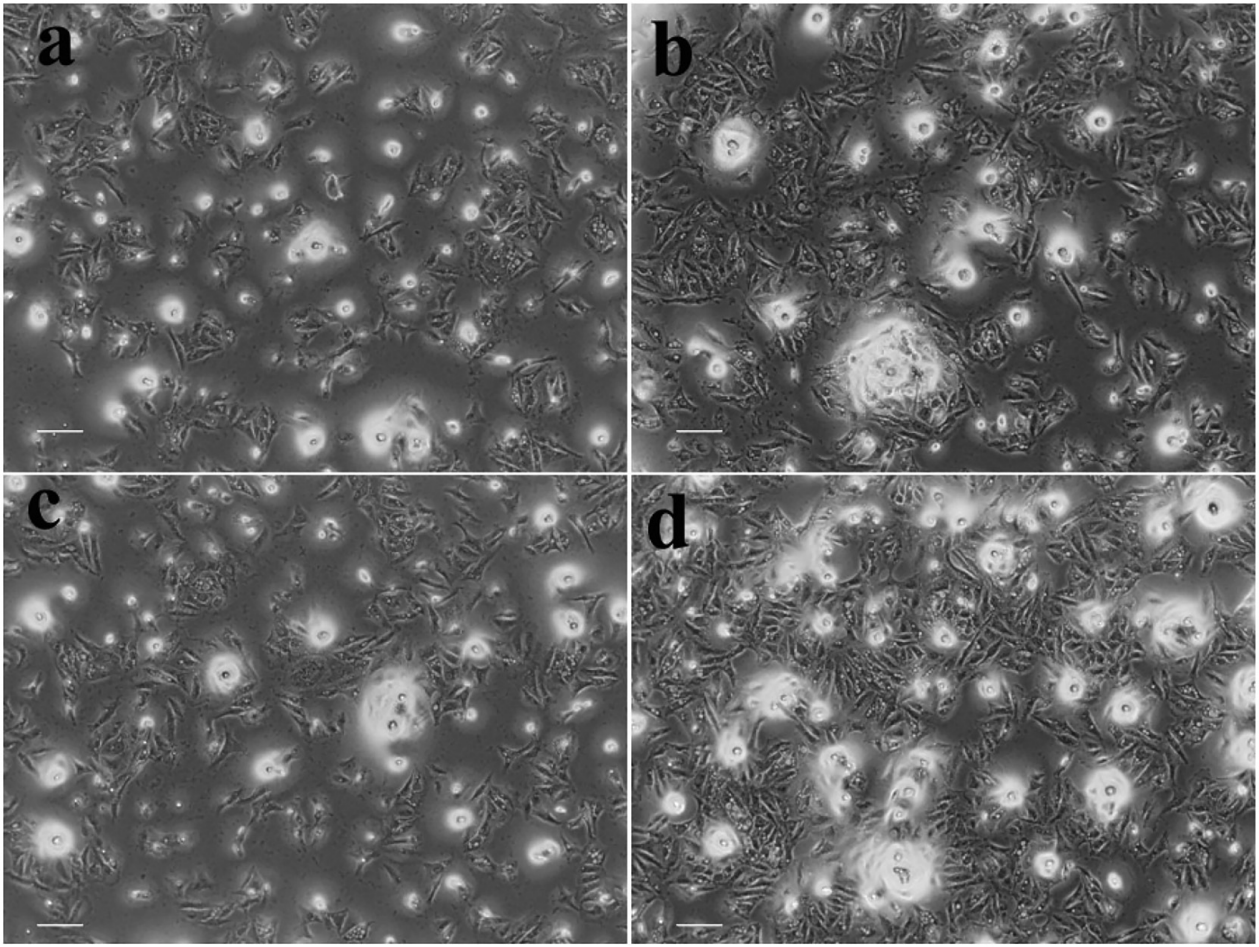
Morphological analysis of SiHa cervical cancer cells treated with *Carica monoica*-coated SPIONs (CM-SPIONs) for 24 h. (a) Untreated control; (b) 25 μg/ml; (c) 50 μg/ml; (d) 100 μg/ml. Images reveal a dose-dependent increase in cell rounding, shrinkage, and detachment, confirming cytotoxic effects consistent with the IC50 value obtained from the MTT assay.

Monitoring morphological alterations in SiHa cervical cancer cells after 24 h of treatment with the IC_50_ concentration of CM-SPIONs elucidates the impact of the nanoparticles on cellular morphology, which may reflect their cytotoxic and therapeutic efficacy (Figure 12). Morphological alterations may provide insights into the mechanism of action of CM-SPIONs. For instance, pronounced cell rounding and detachment may indicate an apoptotic consequence, while substantial cell swelling might signify necrosis (Figure 12b–d). The untreated control cells, meanwhile, did not exhibit any significant impact (Figure 12a). Apoptosis is presumably initiated at lower doses (25 μg/ml), but necrosis becomes more dominant at higher values (50 and 100 μg/ml). Membrane blebbing and cell separation indicate an early apoptotic process, but the observed cell lysis at higher levels indicated necrotic cell death. The interplay of apoptotic and necrotic cell death in reaction to elevated CM-SPION concentrations may indicate the capacity of these nanoparticles to severely impair cellular homeostasis and integrity. Derikvand et al. [60] obtained same results, indicating that bacterial exopolysaccharide-coated magnetic iron oxide nanoparticles induce early death in MCF-7 breast cancer cells.

#### 3.3.5 Ao/EtBr dual staining assay

**Figure 13:**
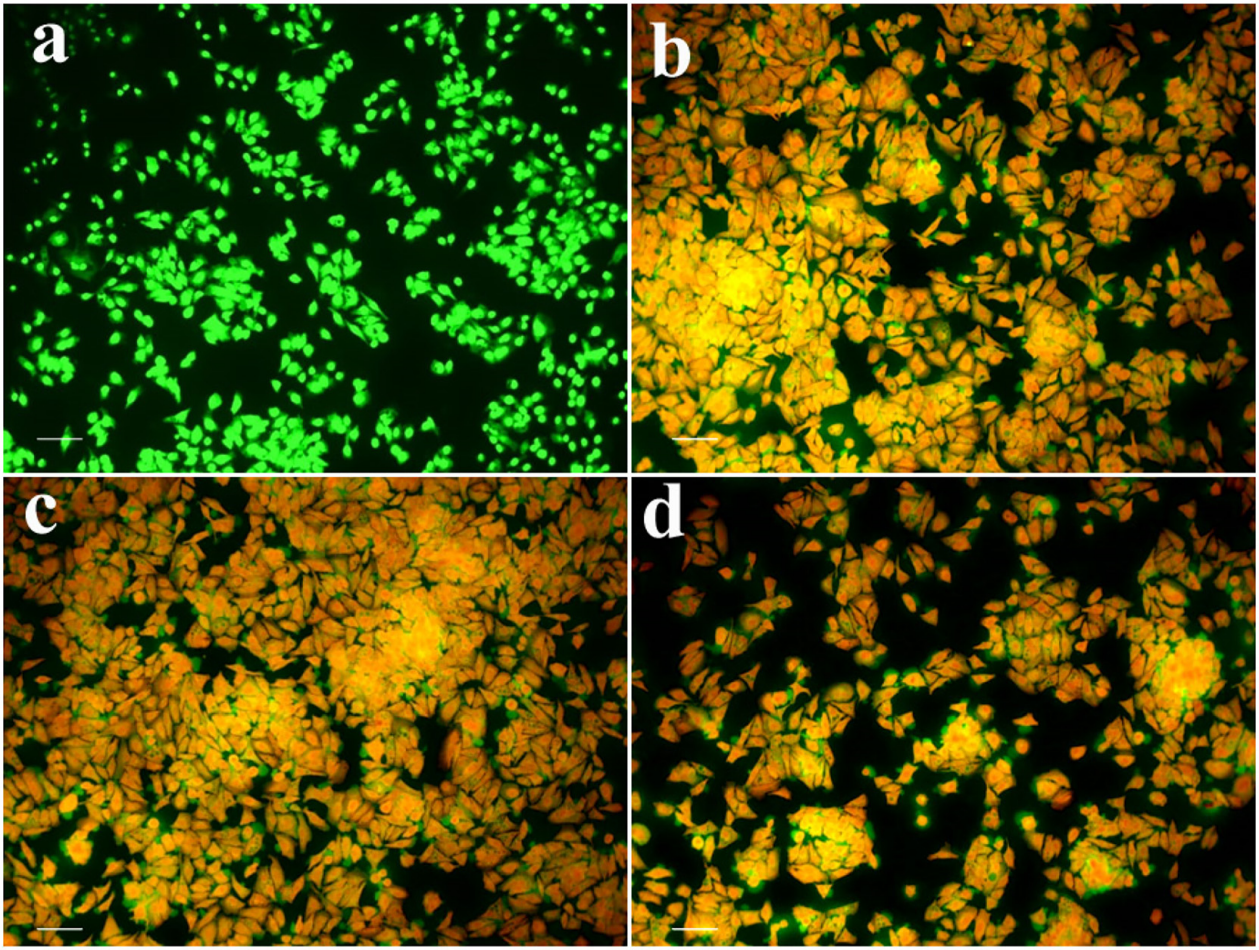
Acridine orange/ethidium bromide (AO/EtBr) dual staining of SiHa cells treated with CM-SPIONs for 24 h. (a) Control; (b) 25 μg/ml; (c) 50 μg/ml; (d) 100 μg/ml. Fluorescence microscopy images show a dose-dependent increase in apoptotic cells (orange/red nuclei) compared to viable cells (green nuclei), confirming induction of programmed cell death.

The results indicated that living cells exhibited green fluorescence, but dead cells had acridine orange fluorescence. Untreated control cells exhibited a substantial quantity of viable cells (Figure 13(a)). SiHa cells subjected to CM-SPIONs demonstrated increased cellular damage, characterised by nuclear shrinkage, membrane blebbing, and nuclear disintegration, manifesting as orangish bodies (Figure 13b–d). By clearly distinguishing between live and dead cells and visualizing apoptotic changes, this method provides important insights into the effectiveness of treatments and the mechanisms of cell death. The dose-dependent increase in apoptotic cells suggests that the cytotoxicity of *CM*-SPIONs is concentration-dependent. This is important for therapeutic applications, as it allows for optimization of the dosage for selective targeting of cancer cells without excessive toxicity to normal tissues [61].

#### 3.3.6 DAPI staining assays

**Figure 14:**
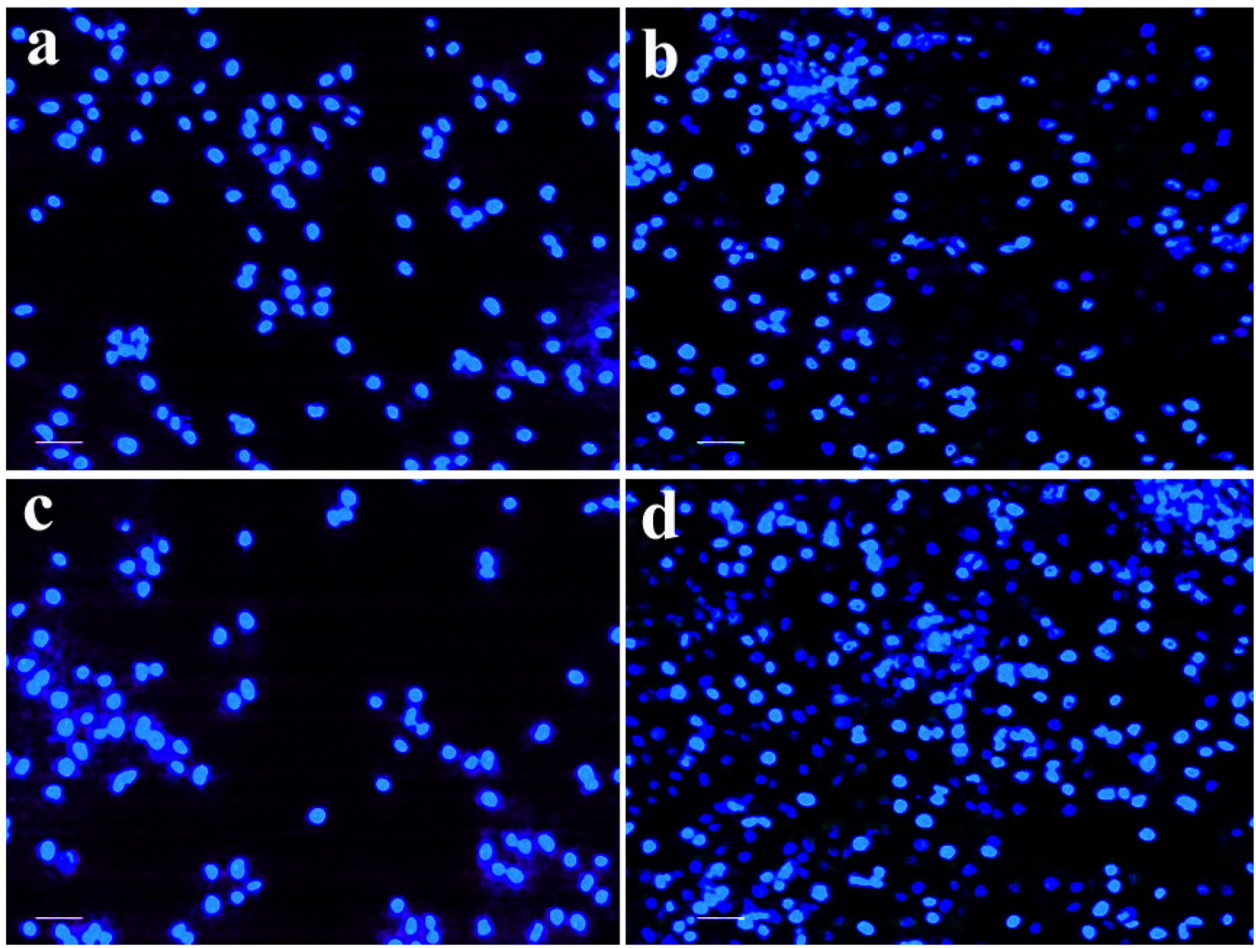
DAPI nuclear staining of SiHa cells following 24-h treatment with CM-SPIONs. (a) Control; (b) 25 μg/ml; (c) 50 μg/ml; (d) 100 μg/ml. Micrographs reveal progressive nuclear condensation and fragmentation at higher doses, providing visual evidence of nanoparticle-induced chromatin damage and apoptosis, consistent with previous viability and staining assays.

Figure 10 shows a fluorescence microscopic picture of cells stained with DAPI after 24 h in the presence and absence of CM-SPIONs. Figure 14a indicates the absence of substantial alterations in the cells, but Bright signals were seen in CM-SPIONs treated cells (Figure 14b–d), indicating the presence of condensed chromatin structures and nuclear fragmentation in SiHa cells. A dose-dependent increase in nuclear fragmentation was observed. At lower concentrations of CM-SPIONs, a moderate amount of nuclear condensation and fragmentation was seen, while at higher concentrations, the percentage of cells with fragmented nuclei increased significantly. This further supports the idea that CM-SPIONs induce a concentration-dependent cytotoxic effect on SiHa cells, causing nuclear damage that ultimately leads to cell death via apoptosis. The nuclear fragmentation seen in DAPI-stained cells likely results from the activation of the apoptotic pathway, which involves the caspase-mediated cleavage of nuclear proteins, including lamins and other structural components of the nuclear envelope [62]. The fragmentation of the DNA into smaller, discrete fragments is a hallmark of late-stage apoptosis, and the significant occurrence of these fragments in the treated group suggests that CM-SPIONs trigger the intrinsic apoptotic pathway in SiHa cells.

#### 3.3.7 ROS measurement

**Figure 15:**
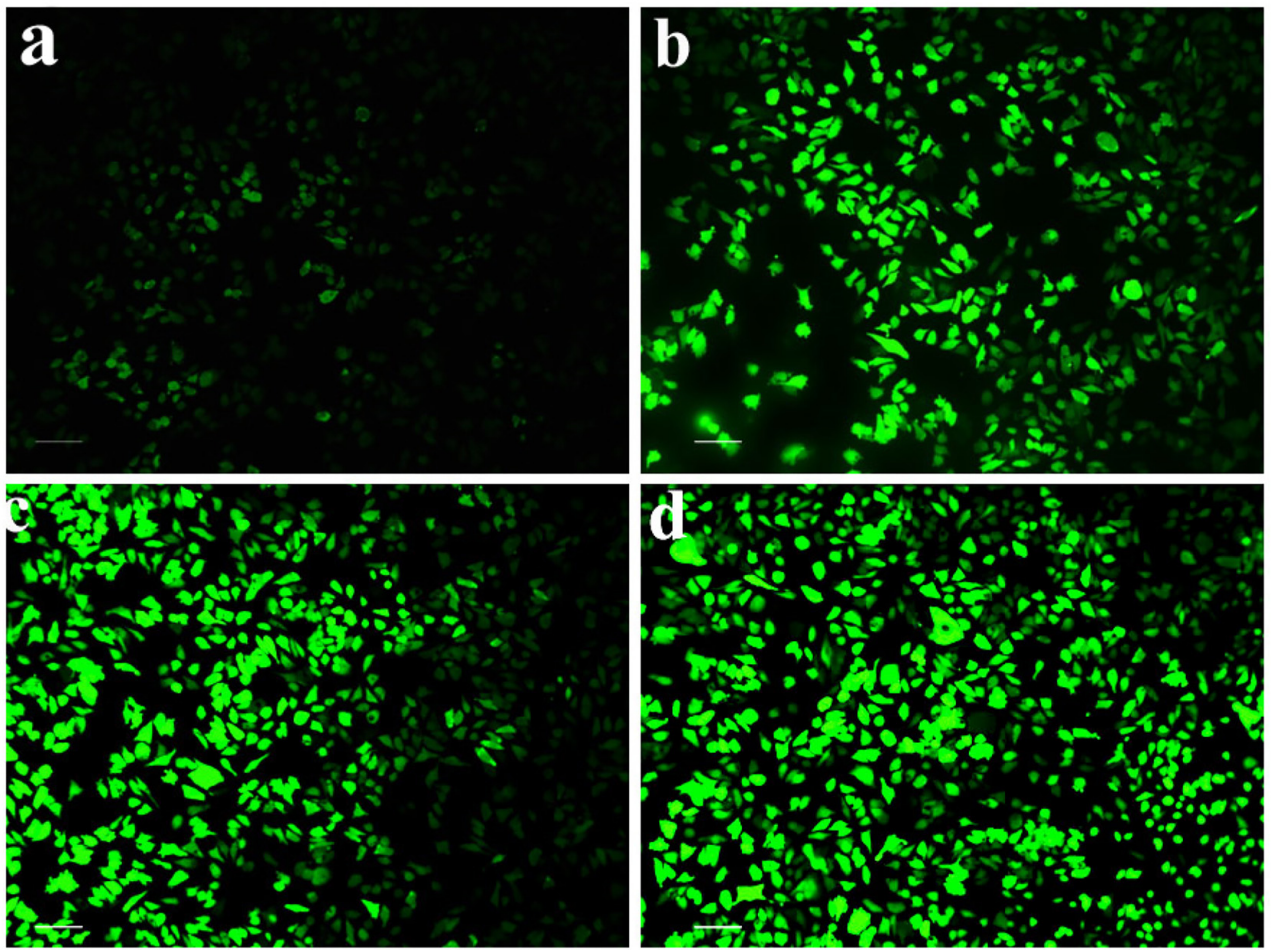
Detection of intracellular reactive oxygen species (ROS) in SiHa cells after 24-h CM-SPIONs treatment using DCFH-DA staining. (a) Control; (b) 25 μg/ml; (c) 50 μg/ml; (d) 100 μg/ml. The dose-dependent increase in green fluorescence intensity indicates elevated oxidative stress, a key mechanism underlying the observed cytotoxicity and apoptosis.

The SiHa cell line treated with CM-SPIONs exhibited increased ROS production in a concentration-dependent manner (Figure 15). In comparison to the untreated control, CM-SPIONs administered to SiHa cells exhibited a dose-dependent increase in green fluorescence intensity, as shown by fluorescence microscopy analysis. The maximum generation of ROS was seen at 100 μg/ml in comparison to the untreated control. Elevated levels of reactive oxygen species (ROS) have been shown to facilitate the demise of cancer cells [63]. The results clearly demonstrate that CM-SPIONs increase ROS generation in a concentration-dependent manner in SiHa cervical cancer cells. Elevated ROS levels are a hallmark of oxidative stress, which is a well-known mechanism of action for many nanoparticle-based therapies. The increased ROS production likely results from the internalization of CM-SPIONs, which may lead to the generation of free radicals and other reactive species, such as hydroxyl radicals, superoxide anions, and hydrogen peroxide. These reactive species can cause cellular damage by oxidizing lipids, proteins, and DNA [64].T

## 4 Conclusions

This study successfully establishes a green, cost-effective, and biocompatible synthesis route for Superparamagnetic Iron Oxide Nanoparticles (SPIONs) using *C. monoica* extract. The outstanding finding of this work is the dual functionality of the synthesized CM-SPIONs, which exhibit both potent broad-spectrum antioxidant activity and selective anticancer properties. The nanoparticles demonstrated exceptional radical scavenging capacity across multiple *in vitro* assays (DPPH, ABTS, FRAP, SOD, reducing power, and H_2_O_2_), with activity exceeding 65 % at 100 μg/ml in key tests. This robust antioxidant profile is directly attributable to the phenolic and flavonoid compounds from the plant extract, which act as both reducing agents during synthesis and as bioactive surface moieties. More significantly, CM-SPIONs exhibited dose-dependent cytotoxicity against human cervical cancer cells (SiHa), inducing apoptosis through mechanisms involving ROS generation, nuclear fragmentation, and mitochondrial pathway activation. The major benefit of this approach lies in its multifunctional therapeutic potential. The same nanoparticles that mitigate oxidative stress a key factor in chronic diseases and cancer progression also directly trigger programmed cell death in malignant cells. This positions CM-SPIONs as promising theranostic agents capable of combining antioxidant protection, anticancer activity, and inherent magnetic targeting in a single platform. Future research should prioritize *in vivo* validation of biocompatibility and efficacy, alongside surface engineering for enhanced tumor targeting. This work paves the way for developing sustainable, plant-based nanotherapeutics that integrate multiple treatment modalities with minimal environmental impact.
